# Examining the impact of medical legal partnerships in improving outcomes on the HIV care continuum: rationale, design and methods

**DOI:** 10.1186/s12913-019-4632-x

**Published:** 2019-11-20

**Authors:** Miguel Muñoz-Laboy, Omar Martinez, Robin Davison, Isa Fernandez

**Affiliations:** 1 Department of Community Health and Social Medicine, School of Medicine, City University of New York, Harris Hall, Room 313B, 160 Convent Avenue, New York, NY 10031 USA; 2 School of Social Work, College of Public Health, Temple University, 1301 Cecil B. Moore Avenue, Ritter Annex, 10G, 5th floor, 505, Philadelphia, PA 19122 USA; 3 College of Osteopathic Medicine, Nova Southeastern University, 3301 College Avenue, Fort Lauderdale, Florida, 33314 USA

**Keywords:** Medical legal partnerships, HIV care continuum, Institutional case study, Intervention mapping legal epidemiology

## Abstract

**Background:**

Over the past two decades, we have seen a nationwide increase in the use of medical-legal partnerships (MLPs) to address health disparities affecting vulnerable populations. These partnerships increase medical teams’ capacity to address social and environmental threats to patients’ health, such as unsafe housing conditions, through partnership with legal professionals. Despite expansions in the use of MLP care models in health care settings, the health outcomes efficacy of MLPs has yet to be examined, particularly for complex chronic conditions such as HIV.

**Methods:**

This on-going mixed-methods study utilizes institutional case study and intervention mapping methodologies to develop an HIV-specific medical legal partnership logic model. Up-to-date, the organizational qualitative data has been collected. The next steps of this study consists of: (1) recruitment of 100 MLP providers through a national survey of clinics, community-based organizations, and hospitals; (2) in-depth interviewing of 50 dyads of MLP service providers and clients living with HIV to gauge the potential large-scale impact of legal partnerships on addressing the unmet needs of this population; and, (3) the development of an MLP intervention model to improve HIV care continuum outcomes using intervention mapping.

**Discussion:**

The proposed study is highly significant because it targets a vulnerable population, PLWHA, and consists of formative and developmental work to investigate the impact of MLPs on health, legal, and psychosocial outcomes within this population. MLPs offer an integrated approach to healthcare delivery that seems promising for meeting the needs of PLWHA, but has yet to be rigorously assessed within this population.

## Background

Four in five physicians in the US agrees that patients’ social and legal needs are as important to address as their medical issues. Eighty-five percent of primary care providers nationwide report that unmet social and legal needs lead directly to inferior health outcomes. Yet 80% of physicians surveyed lacked confidence in their ability to address such needs, impeding their ability to provide quality care [1]. In response to this systemic need, legal epidemiology, defined as the scientific study and deployment of law as a factor in the cause, distribution, and prevention of disease and injury in a population [2], provides a structural framework to better understand the health-harming legal needs of people with chronic illness. Thus, medical-legal partnerships (MLPs) were developed to help medical providers better identify and meet vulnerable patients’ legal needs [3]. Since 1993, MLPs have been established in 294 healthcare institutions in 41 states in the United States. These MLPs provide a multi-faceted approach to health care delivery by integrating legal services and legal advocacy into medicine and health care practices [3–5]. Though the MLP is a promising approach to address the health-harming legal needs of people living with HIV (PLWH), no study prior to our current project has rigorously examined how to build effective MLPs that facilitate positive outcomes in the HIV continuum of care.

In June 2018, the authors were awarded a two-year R21 study funded by the National Institute of Mental Health (#1R21MH115820–01) to develop a culturally-appropriate MLP intervention package to HIV continuum of care outcomes. The specific objectives of this ongoing study are: (1) To identify existing best practices among current MLPs that can be tested, replicated, and scaled-up as evidence-based practices for serving the plurality of PLWH; (2) To assess the effects of the identified MLP practices on: a) appropriately addressing the legal issues through clients’ satisfaction and case outcomes, b) reducing the legally-related psychosocial burdens for PLWH, and, c) increasing positive movement in the HIV continuum of care (including retention in care and viral load suppression) among PLWH. (3) To develop an MLP-comprehensive HIV care diffusion model and its benchmarks of success to achieve positive movement in the HIV continuum of care (including retention in care and viral load suppression) for diverse sectors of PLWH.

## Methods/design

The aim of this paper is to describe the methodology to design a structural intervention to improve HIV care continuum outcomes for people living with HIV. Structural interventions in the field of HIV are usually reported post-hoc with limited or no insights into how they are built and implemented. Drawing on the fields of implementation science in public health and medical anthropology, our study protocol draws on institutional ethnography and intervention mapping methodologies.

### Operationalizing medical-legal partnerships for HIV treatment and care

**Fig. 1:**
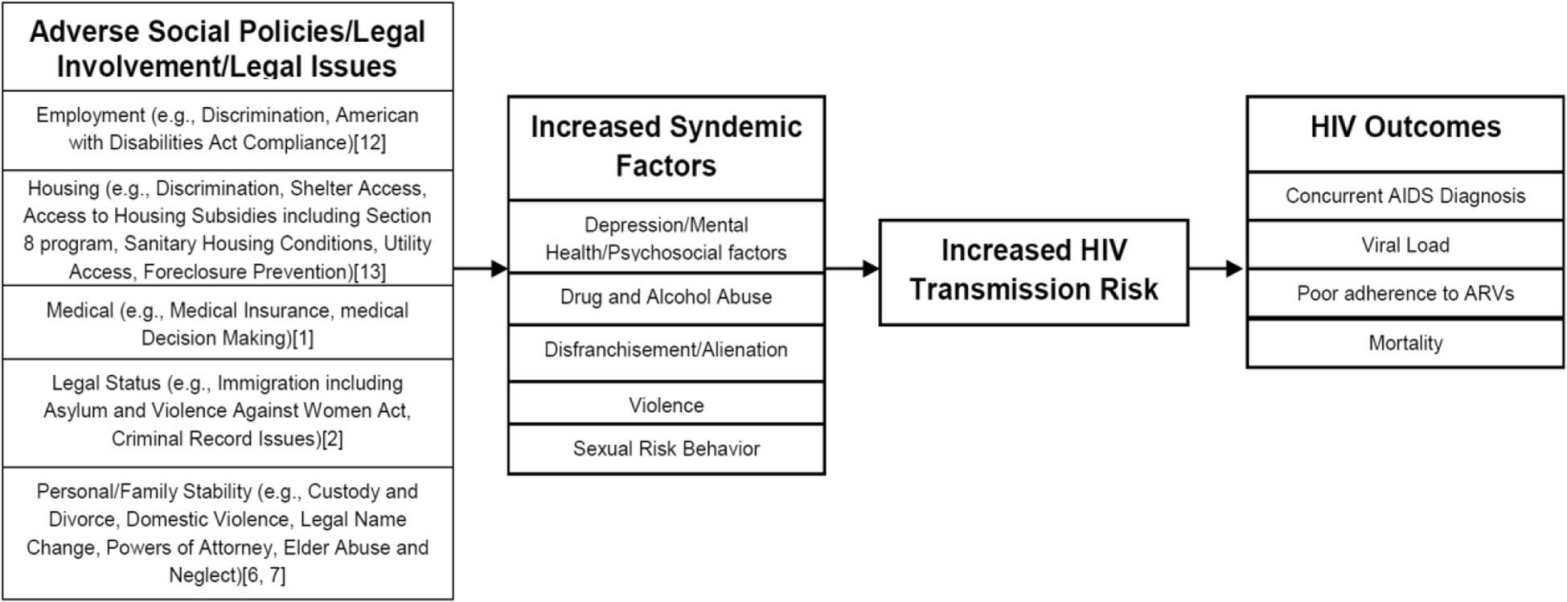
Legal epidemiological framework for MLP with PLWH

HIV treatment and outcome disparities in the US and globally are attributed to syndemic factors, including lack of testing and access to care, discrimination, poor mental health, substance use, violence, and economic hardship [6–9]. Fig. 1 illustrates our legal epidemiological framework that builds on the concepts of structural violence and HIV syndemics [10, 11]. The first column identifies social policies with documented effects on patient wellbeing. We posit that under a legal epidemiological framework, we must address both social and legal needs in order to improve HIV outcomes and reduce excess mortality (i.e., highly preventable deaths below the life expectancy for the individual’s demographic group). MLPs offer a structural, integrated intervention that could tackle these factors. This approach is all the more promising given the potential for HIV status and stigma to exacerbate legal needs. A recent study of people living with HIV/AIDS found that 98% of participants reported at least one legal need within the last year [12]. PLWH face discrimination in their social and work environments because of their HIV status, sexual orientation, and/or substance use [13]. For example, 31 % reported experiencing HIV-based discrimination in employment, housing, and health care settings [12]. Coupled with the medical complications associated with HIV/AIDS, these social issues may have a pernicious impact upon the wellbeing of HIV-affected populations.

Institutional ethnography is an inductive method of inquiry that begins by looking at specific experiences, behaviors, and practices of individuals and then works outward to draw conclusions about the codes, systems, and structures by which they are governed [14, 15]. This “dual focus,” Elizabeth Quinlan notes in “The ‘actualities’ of knowledge work: an institutional ethnography of multi-disciplinary primary health care teams,” is part of what distinguishes it from other types of ethnography (p. 628) [14].

Our institutional ethnographic methodology draws on this dual focus by examining larger social phenomena from the perspective of multiple social actors and institutions. In our study, the social phenomena are the interactions and formal and informal systems governing medical-legal partnerships. The institutions are health care facilities inclusive of hospitals, federally qualified health centers, community health centers, and, HIV/AIDS service organizations. The social actors include: lawyers, legal aid staff, health care and social service providers screening for health-harming legal needs. Using the outward institutional methodological strategy that Smith and others have argued allow us to build institutional case studies of MLPs for PLWH on two main levels: the organizational and community levels [16–18]. The cornerstone of our methodological innovation is combining institutional case studies with a rigorous implementation science methodology in order to build an evidence-based structural intervention to enhance HIV treatment and care.

In order to build our MLP intervention model for PLWH, we are incorporating institutional case studies and Intervention Mapping [19–21]. Intervention mapping is an implementation approach that is based on an ecological framework, that is focused on the multi-level influences on health-promoting behavior, and develops strategies to address them [19]. Intervention mapping has been used to develop programs for a variety of health behaviors, including early detection practices and linking individuals to the continuum of health services [22–25]. This technique specifies processes for integrating theoretical constructs and empirical evidence for the purpose of intervention planning, and helps connect determinants, the identification of proximal behavioral and environmental factors related to a target health outcomes, and the selection of the most appropriate intervention strategies [20]. This approach has been demonstrated to increase the cultural relevance, proper adaptability, and effective uptake of interventions [20].

We began collecting data for our institutional case studies in August 2018 and will continue until May 31, 2019. Our institutional case study methods are divided into three components: 1) organizational qualitative data; 2) organizational-provider-client quantitative data; and, 3) provider-client qualitative data.

### Component 1. Organizational qualitative data

We conducted interviews with 19 MLP key informants, visited four organizations involved in MLPs, and conducted four structured meetings with a Scientific Collaborative Board. Data was collected from July 2018 to January 2019.

Based on the formative work conducted prior to the start of the study and meetings with the leadership at the National Center for Medical Legal Partnerships, we selected an initial sample of six MLPs working with PLWH within 100 miles radius of the authors’ host institution and, through referral, we contacted four additional MLPs. These ten MLPs are located in New York, Pennsylvania, Massachusetts, Hawaii, Wisconsin, Texas, Florida, New Jersey, and Washington, DC.

#### Key informants

Consisted of providers involved in MLPs that provide services to people living with HIV. We conducted interviews with 19 key informants. Key informants represented: lawyers, health providers, social workers, administrators, and researchers with specific expertise and experience in the MLP approach to care. These key informants, selected because of their first-hand knowledge and understanding of the community, provided insight into the issues facing PLWH and the MLPs that serve them and suggested various ways to address these challenges. When possible, we attempted to interview two key informants per MLP. The levels of experience with MLP services for PLWH ranged from 4 years to more than 20 years.

#### Interview guide and procedures

The interview guide was designed by the authors and it contained ten questions and four probing questions. Sample questions included: How would you describe the MLP approach in your agency? What factors contribute to the success of the MLP and why? What are the main challenges associated with MLP implementation? The interviews were conducted in person or by phone, with the authors taking turns asking questions. Unscripted probing and follow-up questions are customary in key informant interviewing. During each interview, the authors took notes independently and subsequently compared recorded informant responses for accuracy. Discrepancies in the notes were clarified within the same week of conducting the informant interviews.

#### Scientific collaborative board

The SCB consisted of a core group of MLP practitioners who are experts in their fields, including clinical care, legal services, administration, and social services. The SCB met to review findings from interviews and site visits and to provide guidance on next steps, including preparation for subsequent NIH grant submissions. The SCB meeting with the full board lasted approximately 2 hours. We also had subsequent individual meetings with SCB members. The authors took notes from the meetings.

#### Data analysis strategy

The analysis for organizational qualitative data followed a comparative approach, viewing data collection and analysis as a single concurrent process in which the method is fluid and evolves as the understanding unfolds from the data, a common approach in institutional ethnography. We first focused on “within” differences among key informants’ MLPs with regard to the provision of HIV services. Once we determined the organizational structure and organizational goals, we identified the various approaches to MLP HIV services and the rationale behind each approach. The researchers identified key components of MLP structure and implementation that were later validated by the SCB. We also gathered field notes and reflections from the sites visits. The authors listed, described, and discussed organizational-level best practices, including practices related to the continua of care and screening strategies for health harming legal needs; agency structure and staffing, including staffing of legal aid providers and operational hours; communication and information sharing among partners within the MLP; and written informational materials for clients and providers, including content of biomedical HIV prevention marketing initiatives. The above analysis was completed by February 15, 2019.

### Component 2. Organizational-provider quantitative data

From February 1 – May 15, 2019 we will conduct an online quantitative survey with 100 health and legal MLP service providers to collect organizational-provider data on MLPs serving PLWH.

#### Sample, eligibility and recruitment

We identified MLP providers through the NCMLP database. The database includes names of existing MLPs, geographic locations (i.e., city and state), and voluntary contact information (e.g., email addresses). A total of 294 MLPs have been registered by the NCMLP. Using a script approved by Temple University IRB, we are soliciting MLP service providers’ participation via email, one 3-min phone call, and one follow-up email. This personal approach will likely increase survey completion. The recruitment phone script and email contain information about the study and benefits associated with completion. We are also creating a moderate study-specific website page, which will include an attractive and interactive introduction and overview of the study, and hypertext-enabled links that allow viewers to contact study staff to inquire about participation, ask questions, and solicit feedback. Those who consent and complete the enrollment process will be asked for contact information (name, e-mail, and phone number) to arrange compensation and/or express interest to be contacted for the dyad in-depth interviews (Component #3). At the conclusion of the survey, participants will be offered a $30 gift card and thanked for their time.

#### Measures

Overall, questions will assess best practices, identify new tools, and serve to develop effective MLP approaches to addressing disparities in HIV treatment and health outcomes. The survey instrument consists of five parts. **Part I** inquires about general MLP information (e.g., geographic location, year of establishment, team structure) and providers’ characteristics (e.g., age, occupation, duties and responsibilities to the MLP). **Part II** focuses on seven standard NCMLP performance measures for the prior 12 months (e.g., proportion of patients who were referred to civil legal aid services and received a legal screening; proportion of patient-clients with health-harming legal needs). **Part III** will focus on identifying any existing services specifically geared towards PLWH and frequency of contextual barriers and facilitators to HIV medical adherence for their client population. **Part IV** focuses on examining health-harming legal, legal challenges affecting patient-clients, barriers to accessing legal services, and strategies for engaging PLWH. **Part V** consists of assessing MLPs level of readiness to adopt, implement and sustain a potential MLP HIV specific model of practice.

#### Completion time

The piloting of the above survey with attorneys, graduate students of health and social services, members of the Scientific Collaborative Board, suggest a completion time between 15 and 20 min. At the end of the survey all participants will be asked if they would be willing to participate in a follow-up research component (Component 3, described below).

#### Data analyses

Using the quantitative data generated, we will conduct two primary analyses. The first level of analysis will focus on identifying what MLPs consider organizational best practices (e.g., innovative practices, evidence-based practices) for HIV treatment and care. To achieve this, we will rank MLPs based on their level of exposure to HIV patients and services provided to HIV patients (answers to questions in Part III) vs. the level of performance as determine by the NCMLP performance measures (Part II of the survey). Using this composite indicator of MLP performance on HIV services, we will conduct a series of basic monomial logistic regression analyses to determine differences between MLPs with high and low performance (i.e., background differences by clientele or geography – Part I measures; differences in barriers and facilitators to HIV medical adherence – Part III measures; and, differences in legal services and challenges - Part IV measures).

The second level of analysis will focus on proximal and distal determinants of health-harming needs. We will also conduct exploratory logistic and linear regression modeling, after assessing that that the appropriate assumptions are met, to test the following hypotheses: H_1_: MLP service structural variables will not be associated with the likelihood of patients with at least one health-harming legal need who were treated by the healthcare organization; H_2_: Providers’ characteristics will not be associated with the likelihood of patients with at least one health-harming legal need who were treated by the healthcare organization; and, H_3_: The frequency of health-harming legal needs will be lower among providers with high exposure to HIV positive clients than their counterparts with lower exposure. If the evidence supports hypotheses 1 and 2, there is no need for tailoring the intervention to reduce the likelihood of health-harming legal needs. If H_1_ and H_2_ are rejected, we will conduct further analyses to determine determinants within MLP structure and provider characteristics that may reduce the likelihood of health-harming legal needs. The findings from testing H_3_ will provide insights into the relevance of exposure to HIV positive cases during the training of MLP providers as part of the development of the HIV MLP diffusion model.

#### Sample size justification

We will recruit a sample of 100 MLP providers divided into four categories by type of function within MLPs: (a) health and social services providers (*n* = 25); medical and clinical providers (*n* = 25); (c) legal services providers; and, (d) administrators (*n* = 25). This sampling approach will allow us to have equal representation of the sectors within MLP practices. A sample size of 100 will allow us to conduct the above proposed exploratory analyses between continuous variables, where there should be at least 10 observations per variable; thus, if conducting an analysis of four independent variables, there should be a minimum sample size of 40. Moreover, before conducting the above exploratory regression analyses, we will examine the variance in each variable to make sure that the basic assumptions for regression analysis are met. We will use the Bonferroni correction, a multiple-comparison correction used when several dependent or independent statistical tests are being performed simultaneously to adjust the alpha coefficient to minimize Type 1 error in our analyses. Because of the exploratory nature of the proposed protocol, it was not appropriate to conduct power sampling calculations. We will conduct post-hoc power calculations to determine the likelihood of Type 2 error to our analyses.

### Component 3. Provider-client qualitative data

Concurrent to component #2 above, we will conduct dyad interviews with MLP providers and PLWH patients/clients. Dyad interviews have been essential elements in the development of empirical data on doctor-patient communication models and interventions to improve provider-patient communication [26–28]. From March 1 to April 30, 2019, we will conduct in-depth interviews with dyads of MLP providers and their PLWH clients.

#### Interview guide

We will use a personal narrative approach to conduct the in-depth interviews with MLP providers and PLWH to allow participants to explain, describe events and experiences in the everyday contexts in which they occur [29–31]. Additional file 1 list the guiding questions for the dyad interviews that will be pilot tested, revised, and approved by the Scientific and Community Collaborative Boards of the study on February 28, 2019.

#### Recruitment sequence, eligibility and sampling for dyad interviews

First, we will recruit 50 MLP providers (25 legal providers and 25 health or social service providers) who complete the online survey under Component #2, based on the answers to the last question in the survey. After consent procedures, MLP providers will participate in the in-depth interview through phone, on-line visual-teleconferencing, and/or in person. MLP providers will recruit PLWH clients into the study by providing the potential participants with a unique ID number (to allow us to trace back the individual’s MLP provider). Fifty PLWH-MLP clients (or recent former clients, within 3 months) will participate in in-depth interviews after completing consent procedures.

We are seeking to recruit a diverse sample of PLWH in terms of sex, age, race/ethnicity, risk to sexual health, and representativeness of a larger population of HIV positive individuals. To be eligible PLWH have to meet the following criteria: 1) Have received MLP services in the past year by MLP provider who participated in the dyad interview; 2) Self-reported being HIV-positive when receiving MLP services; 3) Age 18 or older; 4) Able to speak English and/or Spanish. To achieve a diverse sample of PLWH, we will engage MLP providers in following a theoretically relevant quota sampling frame to select participants [32, 33]. We will ask MLP providers to invite individuals into the dyads interviews according to the following quota sampling frame, until there is a minimum of 2 PLWH per cell: (a) female of reproductive age and/or mothers (*n* = 5), (b) elderly, 60 years and older, sexual minorities (*n* = 5), (c) young and adult sexual minorities (*n* = 5), (d) injecting substance users (*n* = 5), (e) non-injecting substance users (*n* = 5), (f) transgender young and adult men and women (*n* = 5), (g) individuals involved in sex worker (*n* = 5), (h) recent (documented or undocumented) migrants (*n* = 5), (i) ethnic minority individuals (*n* = 5), and, (j) dual diagnosis with HIV and another chronic illness or mental health condition (*n* = 5).

#### Data analyses

Interviews will be transcribed (and translated into English for Spanish speaking interviews) and entered into Dboose, a cloud-based software package specifically designed to handle textual data and its analysis. A codebook will be constructed by two independent coders, including coding families based on the above 6 primary topics. We will take a sample of 10 transcripts, which will be coded collectively by two independent coders, members of the research team, and a research assistant. This will serve to refine the codebook. From this coding exercise, a thematic matrix will be developed. We will conduct 4 analyses using the dyads interviews:

1. *Qualitative impacts of MLPs on HIV care* will focus on identifying illustrative case studies, briefly defined as cases within the dyads narratives that represent the most typical and most deviant, as defined by Yin (2017) within the sample demonstrating the range of variations in MLPs addressing the legal issues through clients’ satisfaction and case outcomes [34].
2. *Concordant-discordant perspectives on reach and effectiveness of MLPs for HIV care* will focus on examining each dyad answer, and classify the answers in terms of concordance to determine the level of agreement on MLP effects in increasing positive movement in the HIV continuum of care [35] among PLWH, and, in reducing the legally-related psychosocial burdens for PLWH.
3. *Confirming and disconfirming cases – cross validation analysis* will allow us to detect MLP cases that demonstrate and support findings and inferences from Components 1 and 2 on the structure and MLP practices, while simultaneously actively searching in the qualitative data cases that contradict, disconfirm and/or provide alternative explanations for researchers’ working inferences on the quantitative analysis under Component 2 [36, 37].
4. *Recommendations for HIV MLP diffusion model.* We will conduct content analysis on domains #4–6 (i.e., 4) Information process exchange; 5) Provider-patient communication: shortcomings and successes; and, 6) Current HIV strategies at MLP), to specify recurrent themes, lessons learned (dos and don’ts), determinants of behavioral change and concrete strategies used and/or experienced by participants to promote positive movement in the HIV continuum of care. The content analysis technique has been used before for identifying different elements of programs and interventions [38]. These will be incorporated into the intervention mapping methodology described below.

In order to conduct the above analyses, the qualitative data will be recoded according to each of the four analyses, thus expanding the original thematic matrix. This second level of advanced coding will be conducted by the investigative team.

### Intervention mapping methods

The institutional case studies and mixed methods data generated through components 1 to 3 will serve as the evidentiary foundation for the design of the MLP intervention. The process of designing interventions is as important and must be as rigorous as the evidence collected. For this reason, we selected using the intervention mapping approach [20]. In a recent NIH sponsored webinar on implementation and dissemination science, the presenter, Dr. María Fernández, from University of Texas (one of the authors of Intervention Mapping), provided empirical evidence for the use of intervention mapping as an innovative methodological approach for building intervention models that can be diffuse and tested [39].

In general terms, intervention mapping consists of using an iterative path from problem identification to problem solving or mitigation. Each of the six steps of intervention mapping comprises several tasks each of which integrates theory and evidence. The completion of all of the steps will serve as the blueprint for the HIV-MLP model based on a foundation of theoretical, empirical and practical information.

Table 1 lists the agenda for the Community Collaborative Board (CCB) [40–42] working meetings based on the six steps of intervention mapping. The CCB will have ten representatives of HIV-affected communities and service providers, including local community leaders and leaders in the provision of HIV legal and other services. They will be recruited through our existing community and MLP organizational networks by extending personal email or mail invitation. The investigative team will prepare the preparatory materials prior to each CCB meeting.

**Table 1:** Intervention mapping CCB meetings for MLP on HIV continuum of care

Intervention Mapping	CCB Working Meeting Topics	Sources of data for IM process from Components 1–3	Schedule
Step1. Conduct a problem analysis, identifying what, if anything, needs to be changed	#1. Logic of the problem – Social services and legal needs of diverse PLWH	• Preliminary logic model of positive change in HIV continuum of care (Component 1) • Descriptive statistics from Part III of online survey (Component 2) • Findings from *Determinants of health-harming needs analyses* (Component 2)	May 15, 2019
Step 2. Create matrices of change objectives by combining performance objectives with determinants of change	#2. Logic of change – Winnable organizational MLP changes to address needs of PLWH	• Findings from *Detecting best practices analysis* (Component 2) • Findings from *Qualitative impacts of MLPs on HIV care* (Component 3)	May 30, 2019
Step 3. Select theory-based intervention methods that match the determinants, and translate these into practical applications	#3. Educational strategies – Concrete learning activities that can have a direct positive impact in increasing integrative HIV care	• Findings from *Detecting best practices analysis* (Component 2) • Findings from *Concordant-discordant perspectives on reach and effectiveness of MLPs for HIV care* (Component 3) • Findings from *Recommendations for HIV MLP diffusion model (*Component 3)	June 15, 2019
Step 4. Integrate methods and the practical applications into an organized program	#4. Building MLP-HIV intervention model package components	Findings from *Recommendations for HIV MLP diffusion model (*Component 3)	June 30, 2019
Step 5. Plan for adoption and sustainability in real-life contexts	#5. Building diffusion strategies for MLP-HIV model	• Descriptive statistics from Parts I and II of survey (Component 2) • Consultations with Scientific Collaborative Board	July 15, 2019
Step 6. Generate an evaluation plan to conduct effect and process evaluations	#6. Building benchmark of success and evaluation indicators for MLP-HIV model	• Findings from Confirming and disconfirming cases – cross validation analysis (Component 3) • Consultations with Scientific Collaborative Board	July 30, 2019

The intervention mapping steps in Table 1 will be accomplished through hybrid online Zoom-in person meetings. The members of the Scientific Collaborative Board will join the last weekend CCB meetings to offer advice on steps 5 and 6. Our intervention mapping approach will continue to be informed by the EPIS implementation science framework stages, particularly step 5, where we will discuss with the CCB how internal and external contextual factors may affect the actual execution of the MLP-HIV model, and factors that could potentially influence the sustainability and scalability of the model.

## Discussion

Intervention mapping has been a successful implementation science methodology for the development of in health-risk reduction and health promoting behaviors. Most of the interventions designed using intervention mapping range in levels of intervention from individual, families, and practitioners to organizational and community levels practices; and in types of conditions ranging from prevention of communicable diseases and early detection of chronic conditions to behavioral management of long-term chronic conditions [19, 20, 22–25]. However, intervention mapping has not been applied to the design of legal interventions in health. Thus, our methodology will allow us to expand the application of intervention mapping to the development of interventions beyond what has been the customary use of intervention mapping. Combining intervention mapping based on data collected through institutional case study methodology can serve as the road map to developing structural interventions in the field of HIV treatment and care, and related fields of highly complex diseases that combine communicable and chronic conditions such as untreated hepatitis C virus (HCV) and untreated substance use disorders.

Because of prior studies design limitations, prior assessments of MLPs cannot isolate the specific effects of MLP services on health outcomes. Most published MLP studies do not measure patient health outcomes or specify the causal connections between MLP and observed effects. To our knowledge, this study is the first U.S. federally funded study on medical legal partnerships. Our methodology is one of the first to empirically document best practices for delivering MLP services to PLWH, outcomes in the HIV care continuum and related psychosocial issues. Our study methodology considers the legal continuum as key component in the delivery of structural interventions. By developing an MLP HIV specific diffusion model, this study will contribute towards the field of HIV structural interventions. The findings from this study will provide insights into the relationships among legal challenges, legal involvement and HIV outcomes for PLWH. The lack of studies of the impact of medical legal partnerships on PLWH, despite their increased use, gives our methodology the potential to be of high public health impact.

## Supplementary information

**Additional file 1.** Data collection instrument.

## Data Availability

Data sharing is not applicable to this article as no datasets were generated or analyzed during the current study.

## Abbreviations

CCB: Community Collaborative Board
EPIS: Explore Preparation Implementation Sustainability framework
HCV: Hepatitis C virus
HIV: Human Immunodeficiency Virus
MLP: Medical-legal partnerships
NCMLP: National Center for Medical-Legal Partnerships
PLWH: People living with HIV
