# Supplementary material for: Examining the impact of medical legal partnerships in improving outcomes on the HIV care continuum: rationale, design and methods
Source: BMC Health Serv Res. 2019 Nov 20;19:849. doi: 10.1186/s12913-019-4632-x (PMC6864982; doi:10.1186/s12913-019-4632-x)
Supplement: Supplementary file 1 — Additional file 1. Data collection instrument. [file 12913_2019_4632_MOESM1_ESM.docx]

Additional file 1

Examining the Impact of Medical Legal Partnerships on

Outcomes for People Living With HIV

**Dyads Open-Ended Interview Guidelines**

**[Draft Version to be revised by Scientific and Community Collaborative Boards]**

Investigators: Omar Martinez, JD, MPH, MS; Miguel Muñoz-Laboy, DrPH; Robin Davison, JD, MPH, Temple University College of Public Health, School of Social Work

**[FOR PROVIDERS:]** Thank you for participating in this interview. I want to remind you that this interview will be audio-recorded and transcribed for qualitative data analysis, but the transcriptions will not include your name or the name of your organization. I have provided you with the list of questions I am going to ask. If at any point something is not clear, please let me know. The larger goal of this study is to develop an MLP intervention protocol specifically for HIV positive populations, therefore we welcome your specific and detailed answers that will provide information we need to understand the components necessary for an effective MLP. [GO TO PAGE 2]

**[FOR CLIENTS:]** Thank you for participating in this interview. I want to remind you that this interview will be audio-recorded and transcribed for qualitative data analysis, but the transcriptions will not include your name or the name of the organization that you receive services from. I have provided you with the list of questions I am going to ask you. If at any point something is not clear, please let me know. The larger goal of this study is to develop an MLP intervention protocol specifically for HIV positive populations, therefore we welcome your specific and detailed answers that will provide information we need to understand the components necessary for an effective MLP. [GO TO PAGE 3]

**Part 1: Providers of Medical-Legal Partnership**

| **Leading question** | **Secondary questions/Probes** | **Interviewer’s notes** |
| --- | --- | --- |
| (1) What brought you to work at MLP? | - When did you begin working in MLP? - What is your role/responsibilities? |  |
| (2) What services does your MLP provide? How do you determine which services each individual needs? | - Screening (who is screened, screening tool, screen in or screen out) - Direct (on-site) v indirect (off-site through referral) - Is there a difference in outcomes based on location of services? - Are there services that are specifically intended for HIV positive individuals? - Timeline of services |  |
| (3) How does MLP staff communicate internally? With patients/clients? | - What record keeping method do you use? - How do you handle confidentiality among MLP staff? - Methods of communication with patients/clients, frequency of communication (particularly with lawyer-client communication) - Challenges? What would you change? |  |
| (4) How well does the MLP address its patients’/clients’ needs? Any intervention strategies specifically designed to improve HIV medication adherence and engagement in care? What would you change? | - What kinds of general needs and examples - HIV adherence and engagement in care and examples - Suggestions? |  |
| (5) What is the MLPs impact on the overall health of HIV positive individuals? Taking a critical view of the MLP, what are the areas of the HIV care that you would improve? | - Medical care, ART, ID of legal and social service issues - Focus on at least one example and identify concrete illustrations of overall health effects - F/U question: How do these issues affect their HIV medical adherence and viral load suppression? - F/U: What are the barriers to health for your HIV positive patients? |  |
| (6) MLP approach in practice | - What is most challenging about the MLP and why? - What are the most successful aspects of MLP and why? - What would you change and why? |  |
| (7) Does MLP collect data and assess impact of the MLP on patients/clients? Business? HR? | - Patient/Client: Improved health? Quality of life? - Business impact: Cost-benefit analysis, #ER visits, cost of care, # of people virally suppressed - HR: Job satisfaction, turnover, other measures? |  |
| (8) What have been major successes with PLWH patients/clients? What measurable behavioral changes were observed? | - Measurable behavioral changes, are changes that can be observed or quantified. - Make sure to list as many as possible; if none, why do you think there haven’t been any? Do you have suggestions? |  |
| (9) Internal MLP operations | - How open are MLP administrators to making improvements and responding to complaints/suggestions for MLP improvement? - What training do MLP (medical, legal, social service) staff receive? - What would you make any changes? |  |

**Part 2: Clients of Medical-Legal Partnership**

| **Leading question** | **Secondary questions/Probes** | **Interviewer’s notes** |
| --- | --- | --- |
| (1) What brought you to seek care at MLP? What were/are the barriers that make it difficult for you to get health/medical care? | - How did you become connected to MLP? - First connection through medical or legal? - Prior MLP experiences (if any) - Top reasons to attend current MLP - Length of time receiving services from MLP |  |
| (2a) What services have you received/do you receive through MLP? Where do you receive these services? | - Medical, legal, social, other services - On-site, off-site, other? |  |
| (2b) What were the issues you were dealing with that led you to become connected to these services? How were these issues identified and by whom? | - Identify legal/social issues past and present - How were these issues identified? (Screening? Self-reported?) |  |
| (3a) What is your understanding about how information is shared about you among MLP partners who are involved in your care? | - Please explain. - Do you recall signing a form consenting to the sharing of your info? - Do you feel comfortable with your information being shared among MLP staff? |  |
| (3b) Please describe your communication with the MLP medical staff? MLP legal staff? | - F/U question: mode of communication, frequency, ease of access - Any difficulties |  |
| (4a) How long did it take for you to become connected to legal support to help you with your issue(s)? Social services support? Other support? | - Determine time it took for legal appointment, social services appointment, etc. |  |
| (4b) Were the legal/social services you received helpful? How? | - Explain result of legal services - Explain result of social services |  |
| (4c) Have you had any problems getting services through the MLP? | - Contact person, wait time for appointment or services, etc. |  |
| (4d) Are you dealing with any issues that have not been addressed by the MLP? | - What issues? What has prevented these issues from being addressed? |  |
| (5) How did the MLP care you received affect your health? What would you change? | - Has your overall health changed since you began receiving support through the MLP? - Has your HIV care /medication adherence changed since you began receiving support through the MLP? - Was there a time when your needs weren’t addressed effectively? Please explain. |  |
| (6) Beyond changes to your health, has anything else in your life changed a result of your involvement with MLP? | - Enrolled in benefit programs, financial stability, quality of life, etc. - What has been the most helpful or impactful part of your engagement with the MLP and why? - What has been the most challenging part of your engagement with the MLP and why? - What would you change about the MLP and why? |  |
| (8) Has your level of engagement with the community changed since you began receiving support from the MLP? | - Examples |  |
| (9) Is there someone at the MLP who is available for you to speak with about any problems you are experiencing with the MLP? | - Elicit information on openness, responsiveness, ongoing improvement |  |
